# Optimal treatment strategy of fremanezumab in migraine prevention: a systematic review with network meta-analysis of randomized clinical trials

**DOI:** 10.1038/s41598-020-75602-8

**Published:** 2020-10-29

**Authors:** I-Hsin Huang, Po-Chien Wu, Ya-Han Lee, Yi-No Kang

**Affiliations:** 1 Department of General Medicine, Taipei Medical University Hospital, Taipei, Taiwan; 2 Department of Medical Education, Taipei Veterans General Hospital, Taipei, Taiwan; 3 Department of Pharmacy, Wan Fang Hospital, Taipei Medical University, Taipei, Taiwan; 4 Department of Clinical Pharmacy, School of Pharmacy, Taipei Medical University, Taipei, Taiwan; 5 Evidence-Based Medicine Center, Wan Fang Hospital, Taipei Medical University, No. 111, Section 3, Xing-Long Road, Taipei, 11696 Taiwan; 6 Research Center of Big Data and Meta-Analysis, Wan Fang Hospital, Taipei Medical University, Taipei, Taiwan; 7 Cochrane Taiwan, Taipei Medical University, Taipei, Taiwan; 8 Institute of Health Policy and Management, College of Public Health, National Taiwan University, Taipei, Taiwan

**Keywords:** Drug delivery, Drug safety, Migraine

## Abstract

Identifying the optimal fremanezumab treatment strategy is crucial in treating patients with migraines. The optimal strategy was investigated by assessing the cumulative 50% reduction rate (50%CRR), cumulative 75% reduction rate (75%CRR), reduction in the number of migraine days, treatment-related adverse events, and serious adverse events in patients treated with fremanezumab 225 mg monthly (225 mg), 675 mg monthly (675 mg), 900 mg monthly (900 mg), a single high dose of 675 mg (S675mg), 675 mg at baseline with 225 mg monthly (675/225 mg), and placebo. Biomedical databases were searched for randomized controlled trials on this topic, and data were individually extracted. Risk ratios and mean differences were used to present the pooled results. The surface under the cumulative ranking curve (SUCRA) was used to determine the effects of the medication strategies of fremanezumab. Five trials (n = 3404) were used to form a six-node network meta-analysis. All fremanezumab medication strategies displayed significantly higher cumulative 50% reduction rates than the placebo. The SUCRA revealed that treatment with 675 mg yielded the highest 50%CRR value (mean rank = 2.5). S675 mg was the only treatment with significantly higher 75%CRR reduction rate than placebo, whereas the SUCRA for 225 mg displayed the highest mean rank (2.2). Moreover, 225 mg (mean rank = 2.2) and S675 mg (mean rank = 2.2) presented lower probabilities of serious adverse events. Collectively, S675mg and 225 mg exhibited the optimal balance between efficacy and safety within three months. Long-term efficacy and safety remain unclear, and future studies should further evaluate the long-term outcomes.

## Introduction

Migraines are a highly common and disabling neurological disorder, characterized by headache, throbbing, aura, nausea, vomiting, photophobia, and cognitive dysfunction^1,2^. The development of treatments for migraine has been a major focus of research over the past decade, and curative treatment findings have been unveiled^3^. Studies have discovered that the calcitonin gene-related peptide (CGRP), a multifunctional neuropeptide that plays an important role in the pathogenesis of migraine, may be a promising target for migraine treatment^4,5^.

Fremanezumab (TEV-48125), a fully humanized monoclonal antibody, was the first medication approved by the Food and Drug Administration that selectively binds to CGRP and prevents its ligation to its receptor. The engineered humanized IgG2k structure reduces immunogenic-related interaction with the immune system, which broadens its applications^6^. Fremanezumab targets migraines by connecting to CGRP ligand and obstructing binding to the receptor, thereby inhibiting signal transduction involved in migraine episodes^7^. Moreover, the safety, tolerability, and efficacy of fremanezumab have been studied in numerous randomized controlled trials (RCTs) in all anti-CGRP antibodies. The pharmacokinetic effects are also involved in fremanezumab efficacy. The time to maximum concentration of fremanezumab administered subcutaneously is approximately 5–7 days, and bioavailability was reported at 65.8%^8^. Furthermore, fremanezumab is degraded by enzymatic proteolysis into small peptides and amino acids in the absence of metabolism by liver enzymes, which may prevent drug–drug interactions. Fremanezumab has a long half-life of 31 days and reaches the steady state at approximately 6 months. Therefore, doses were only to be administered at intervals of 1–3 months in clinical trials^9–13^. Moreover, higher doses of humanized monoclonal antibodies have been proposed to saturate cell membrane targets responsible for elimination, causing nonproportional increases in mAb serum concentration^14^. This nonlinear feature of fremanezumab was tested using a two-compartment model with first-order absorption and elimination^8^. However, the effects of this exposure–response relationship on efficacy and safety require further evaluation.

A review focusing on the use of fremanezumab in treating migraine concluded that fremanezumab exhibited higher tolerability and prophylactic effects for episodic and chronic migraine compared with other medications^15^. Furthermore, most meta-analyses have concluded that anti-CGRP antibodies are an effective and well-tolerated preventive treatment for chronic or episodic migraines^16–19^. However, no study has provided a robust conclusion regarding the clinical medication strategy that provides optimal efficacy. In our previous systematic review and meta-analysis, we evidenced that treatment with anti-CGRP antibodies significantly improved the response rate compared with placebo, in terms of the reduction in the number of migraine days, with a slightly decreasing trend observed during the 3-month treatment interval^16^. Heterogeneities were reduced using a subgroup analysis of four anti-CGRP medications, except in the results of fremanezumab. The high heterogeneity of the fremanezumab analysis may reflect the variation in medication strategies, which was a limitation of the past head-to-head meta-analysis. The optimal treatment strategy of fremanezumab in treating patients with migraine should be identified to improve migraine management in clinical practice^20^.

In the present study, we investigated different fremanezumab medication strategies for the treatment of migraines, thus extending the analysis of RCTs to enable use of this agent in a clinical setting. Furthermore, we analyzed the occurrence of adverse events for each medication strategy.

## Results

**Figure 1:**
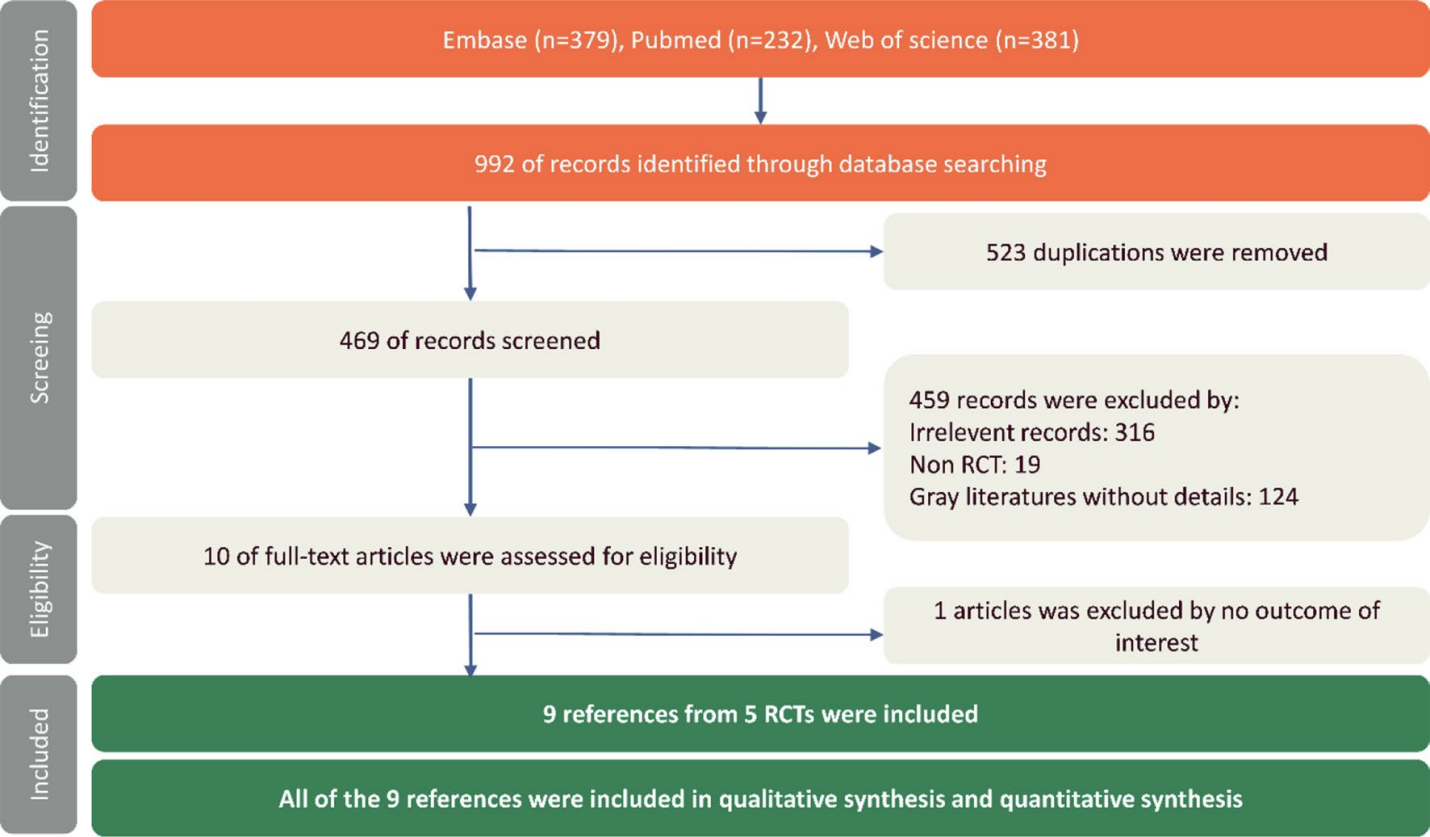
Flow diagram of study selection. RCT, randomized clinical trial.

Our database search strategy yielded a total of 992 references. After 523 duplicated references and 459 irrelevant references were excluded, we accessed the full texts of the remaining ten references for review. A total of nine articles from five RCTs were included in our synthesis (Fig. 1).

### Characteristics and quality of included studies

**Figure 2:**
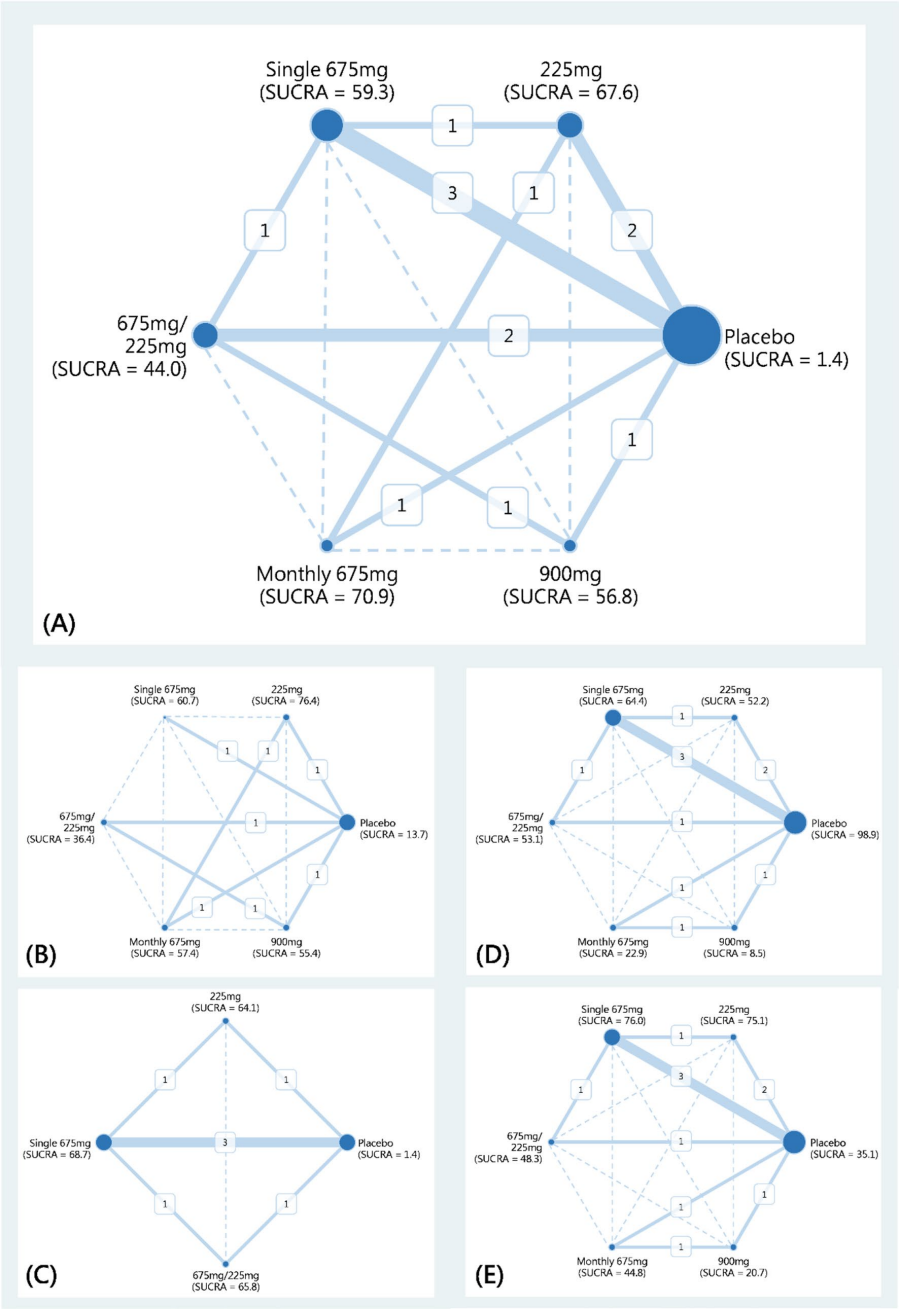
Network plots of medication strategies of fremanezumab in (**A**) cumulative 50% reduction rate of migraine, (**B**) cumulative 75% reduction rate of migraine, (**C**) reduction of migraine days, (**D**) treatment related adverse event, and (**E**) serious adverse event.

The five RCTs investigated a total of 3,404 patients with migraines from Europe and North America between January 2014 and July 2018. The mean age of patients in each study ranged from 40 to 46.8 years. The studies included 483 men (14.19%) and 2921 women (85.81%)^9,21–28^. Table 1 presents the demographic information, and Supplement File S1 presents the quality of the RCTs. These trials formed networks for the cumulative 50% reduction rate (Fig. 2A), cumulative 75% reduction rate (Fig. 2B), reduction in the number of migraine days per month (Fig. 2C), treatment-related adverse events (Fig. 2D), and serious adverse events (Fig. 2E).

**Table 1:** Characteristics of the included RCTs.

Trial	Area	Trial period	Medication	Male/female	Age	Type of migraine
NCT02629861^23^	North America and Europe	2016 to 2017	Fremanezumab 225 mg monthly	46/244	42.9	Episodic
			Fremanezumab 675 mg single high dose	40/251	41.1	
			Placebo	47/247	41.3	
NCT02621931^25^	North America	2016 to 2017	Fremanezumab 675 mg + 225 mg*2	49/330	40.6	Chronic
			Fremanezumab 675 mg single high dose	45/331	42.0	
			Placebo	45/330	41.4	
NCT02021773^9,21,22,26^	North America	2014	Fremanezumab 900 mg*3	12/75	41.5	Chronic
			Fremanezumab 675 + 225 mg*2	12/76	40.0	
			Placebo	13/76	40.7	
NCT02025556^52^	North America	2014 to 2015	Fremanezumab 675 mg*3	15/82	40.7	Episodic
			Fremanezumab 225 mg*3	9/87	40.8	
			Placebo	12/92	42.0	
NCT03308968^27^	North America and Europe	2017 to 2018	Fremanezumab 225 mg*3/675 mg + 225 mg*2	45/238	45.9	Episodic or chronic
			Fremanezumab 675 mg single high dose	47/229	45.8	
			Placebo	46/233	46.8

### Efficacy

**Figure 3:**
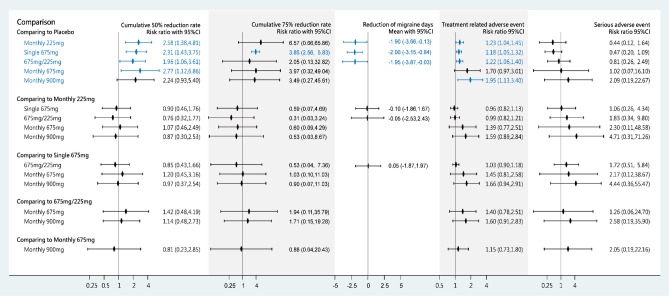
Forest plots of medication strategies of fremanezumab in cumulative 50% reduction rate of migraine, cumulative 75% reduction rate of migraine, reduction of migraine days, treatment related adverse event, and serious adverse event. Blue letters, diamonds, and confidence interval lines refer to statistical significance.

Studies have reported that the efficacy of fremanezumab is mainly indicated in the cumulative 50% reduction rate, cumulative 75% reduction rate, and reduction in the number of migraine days per month. Five of the trials reported data regarding the cumulative 50% reduction rate within 3 months^9,21–28^. The treatment period was 12 weeks in all trials and the RCTs investigated five medication strategies using fremanezumab: 225 mg monthly (225 mg), 675 mg monthly (675 mg), 900 mg monthly (900 mg), a single high dose of 675 mg (S675 mg), or 675 mg at baseline plus 225 mg per month (675/225 mg). The available data indicated that fremanezumab 675 mg (risk ratio [RR] 2.77, 95% confidence interval [CI] 1.12–6.86), 225 mg (RR 2.58, 95%CI 1.38–4.81), S675 mg (RR 2.31, 95%CI 1.43–3.75), 900 mg (RR 2.24, 95%CI 0.93–5.40), and 675/225 mg (RR 1.96, 95%CI 1.06–3.61) significantly increased cumulative 50% reduction rates compared with the placebo (Fig. 3). The surface under the cumulative ranking curve (SUCRA) analysis revealed that monthly treatment with 675 mg fremanezumab yielded the highest probability and mean rank (mean rank = 2.5; SUCRA = 70.9), followed by 225 mg (SUCRA = 67.6), S675 mg (SUCRA = 59.3), 900 mg (SUCRA = 56.8), 675/225 mg (SUCRA = 44.0), and placebo (SUCRA = 1.4) (Supplementary File S2). That is, medication strategies with equal monthly dosages resulted in higher 50% reduction rates than combination dosages. Loop inconsistency (*P* = 0.763; Supplementary File S3) and small study effect (*P* = 0.385; Supplementary File S4) were not observed in the pooled estimates.

Three trials (n = 1110) reported the cumulative 75% reduction rate during the 3-month treatment. In the pooled estimates, S675 mg (RR 3.86, 95%CI 2.56–5.83) was the only treatment that displayed significantly higher cumulative 75% reduction rates than placebo treatment. However, the SUCRA analysis demonstrated that 225 mg fremanezumab had the highest value (76.4) and mean rank (2.2) (Supplementary File S5). Loop inconsistency (*P* = 0.666; Supplementary File S6) was not observed in the pooled estimates, and the funnel plot did not display asymmetry in the network meta-analysis of the cumulative 75% reduction rate (Supplementary File S7). Egger’s test failed to detect small study effects because of insufficient formation among comparisons. However, Begg–Mazumdar method was used as an alternative and did not detect a significant small study effect in the pooled estimate (z =  − 0.11; *P* = 1.00).

Three of the five trials (n = 2540) reported reductions in the number of migraine days after treatment with fremanezumab 225 mg monthly, S675 mg, 675/225 mg, or placebo (Fig. 3). However, the trials only used these medication strategies. Therefore, our consistency model of the reduction in the number of migraine days did not include fremanezumab 675 mg and 900 mg. The pooled estimates revealed that treatment with S675 mg (mean difference [MD] =  − 2.00, 95%CI − 3.15 to − 0.84), 675/225 mg (MD =  − 1.95, 95%CI − 3.87 to − 0.03), and 225 mg of fremanezumab (MD =  − 1.90, 95%CI − 3.66 to − 0.13) caused significant reductions in the number of migraine days compared with placebo. Similar trends were observed in the mean rank and SUCRA analysis (Supplementary File S8). Loop inconsistency (*P* = 0.961; Supplementary File S9) and small study effect (*P* = 0.723; Supplementary File S10) were not observed in the pooled estimate of the reduction in the number of migraine days.

### Safety

To assess the safety of fremanezumab, we investigated treatment-related adverse events and serious adverse events. Four of the five RCTs (n = 2801) reported treatment-related adverse events. The pooled estimates of treatment-related adverse events did not all reach clinical significance. However, 900 mg (RR 1.95, 95%CI 1.33–3.40), 225 mg (RR 1.23, 95%CI 1.04–1.45), 675/225 mg (RR 1.22, 95%CI 1.06–1.40), and S675 mg (RR 1.18, 95%CI 1.05–1.32) resulted in significantly higher rates of treatment-related adverse events compared with placebo. The effect size of 675 mg of fremanezumab monthly was the second most favorable, and this treatment did not result in a significantly higher rate of treatment-related adverse events compared with placebo. The SUCRA analysis indicated that S675 mg yielded the highest probability of reducing the risk of treatment-related adverse events (mean rank = 2.8; SUCRA = 64.4), whereas 900 mg of fremanezumab monthly displayed the lowest value (mean rank = 5.6; SUCRA = 8.5; Supplementary File S11). Loop inconsistency (*P* = 0.551; Supplementary File S12) and small study effect (*P* = 0.606; Supplementary File S13) were not observed in the pooled estimates of treatment-related adverse events.

Serious adverse events were reported in all the included RCTs (Fig. 3). The pooled estimate did not reveal significant differences among treatments. However, the SUCRA analysis demonstrated that S675 mg (mean rank = 2.2; SUCRA = 76.0) and 225 mg (mean rank = 2.2; SUCRA = 75.1) had similar mean ranks and SUCRA values, followed by 675/225 mg (SUCRA = 48.3), 675 mg (SUCRA = 44.8), and 900 mg (SUCRA = 20.7) (Supplementary File S14). Loop inconsistency (*P* = 0.556; Supplementary File S15) and small study effect (*P* = 0.706; Supplementary File S16) were not observed in the network meta-analysis of serious adverse events.

### SUCRA cluster

**Figure 4:**
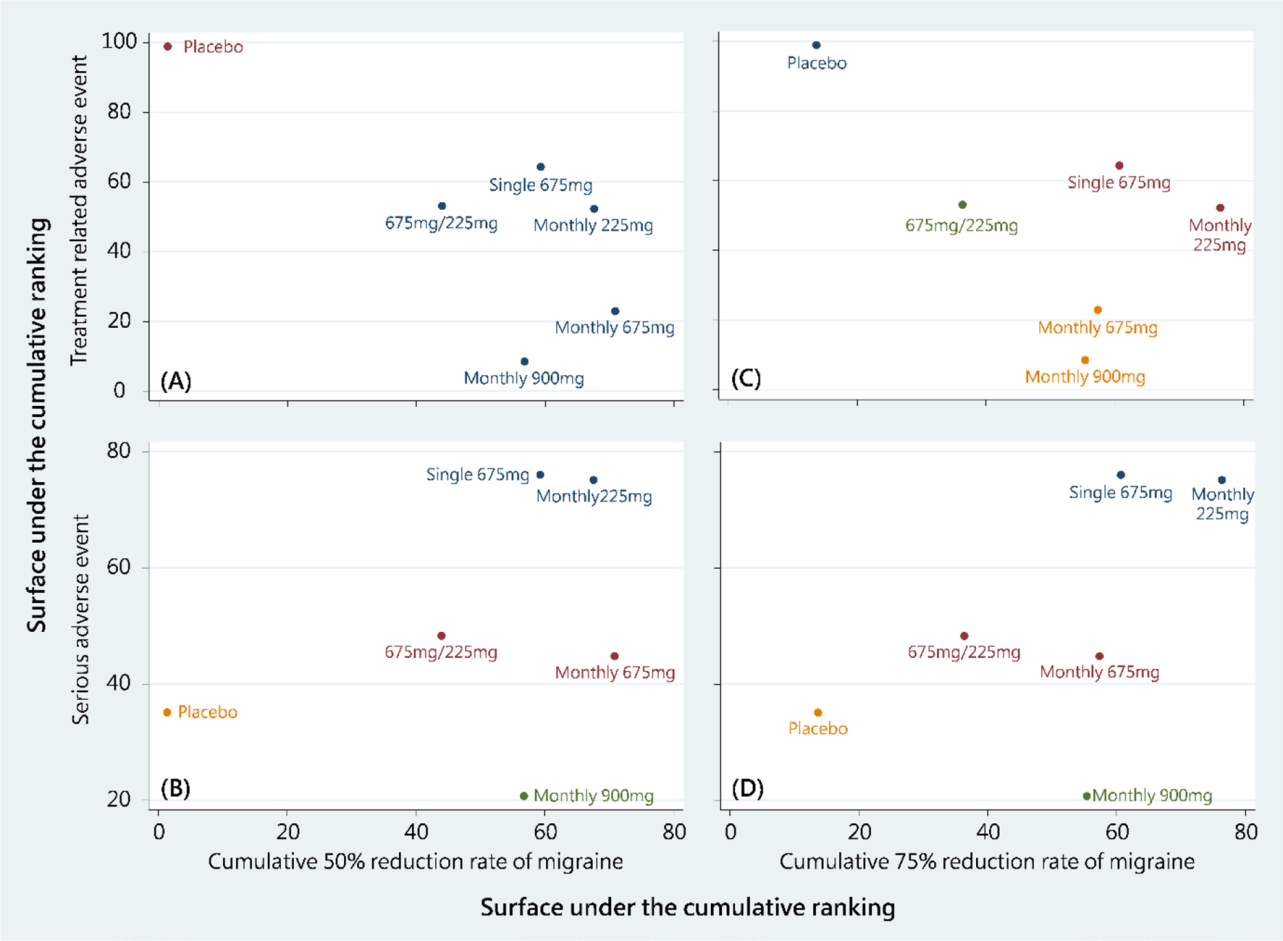
Surface under the cumulative ranking cluster plots of (**A**) cumulative 50% reduction rate of migraine and treatment related adverse event, (**B**) cumulative 50% reduction rate of migraine and serious adverse event, (**C**) cumulative 75% reduction rate of migraine and treatment related adverse event, and (**D**) cumulative 75% reduction rate of migraine and serious adverse event. A color refers to a cluster of treatments.

Cluster plots of the cumulative 50% reduction rate and treatment-related adverse events revealed ambiguous patterns (Fig. 4). The cluster plots of the cumulative 50% reduction rates with serious adverse events, cumulative 75% reduction rate, and treatment-related adverse events indicated that fremanezumab 225 mg and S675 mg exhibited the most favorable balance. Similar trends were observed in the cluster plots of the cumulative 75% reduction rates, treatment-related adverse events, and serious adverse events. Fremanezumab 900 mg and 675/225 mg appeared relatively unbalanced in terms of efficacy and safety. Therefore, these two medication strategies should be administered with caution for the treatment of patients with migraine.

## Discussion

In the present analysis, all fremanezumab medication strategies displayed significantly higher 50% reduction rates than placebo treatments. Monthly treatment with 675 mg fremanezumab displayed the highest 50% reduction rate compared with the other medication strategies and the placebo, whereas fremanezumab S675 mg and monthly 225 mg had a higher probability to be the most favorable treatments in terms of 75% reduction rate. Although the differences between the medication strategies did not reach statistical significance, the higher efficacy of the monthly equal dosages appears more favorable. The efficacy of fremanezumab 900 mg is contentious because only 86 patients were treated with this medication strategy.

Regardless of the overall efficacy of fremanezumab, rapid onset of treatment effect has been proven^29^. Early treatment may induce more favorable clinical outcomes. Therefore, fremanezumab may improve patient compliance and reduce the use of acute headache medications^30^. With the dosage effect of fremanezumab reported in this study, further studies evaluating the early treatment effects of different treatment strategies are warranted.

The pooled results indicated that fremanezumab significantly reduced the number of migraine days per month compared with placebo. However, this study endpoint may not accurately represent the efficacy of the interventions. Patients were enrolled with variable baseline migraine days. Therefore, the same number of reductions in the number of migraine days per month were recorded as different percentage rates.

The efficacy of the interventions was related to the different types of migraines, namely episodic or chronic migraines. In most trials, chronic migraines were treated with higher medication dosages than episodic migraines. However, our analysis revealed that an equal monthly dosage was more effective than varying dosages, which indicated that the higher dosage strategy may be inappropriate. Medications with an equal monthly dosage may offer efficacy equivalent to that observed with the use of different dosages and a consequently larger total amount of medication. Therefore, a consistent lower dosage may be more effective for treating patients with chronic migraines.

Concerns have been raised regarding the clinical application of fremanezumab, despite its clinical efficacy, safety, and tolerability^31,32^. The most common reported adverse effects of fremanezumab with a high incidence rate is a local injection site reaction, which may be related to the subcutaneous injection route of administration^33^. Infusion reactions typically occur within 1–2 h of starting an infusion. Subcutaneous treatment administration can cause injection site reactions, including swelling, itching, redness, pain, or even anaphylaxis^34^. Mild reactions are common. However, serious side effects are the principal concern in clinical practice because tolerability has a profound effect on patient adherence and compliance^35^. The control of migraine and the intolerable adverse events may affect patients’ disability-adjusted life-years. Therefore, selecting the optimal dosage and treatment strategy by determining the balance of the risk–benefit profile is crucial^29,36^.

The analysis of serious adverse events revealed that a higher total dosage was associated with a higher occurrence of serious adverse events, although the results did not reach statistical significance. Notably, the placebo group exhibited a higher risk of serious adverse events than the 225 mg, S675 mg, and 675/225 mg treatment groups. This discrepancy may be attributed to the varied definitions of serious adverse events in different RCTs. Furthermore, some serious adverse events were not related to treatment. Further studies are warranted to evaluate serious treatment-related events caused by fremanezumab use.

Numerous studies have provided meta-analyses of the pooled results of all anti-CGRP medications. However, only one study has focused on the efficacy and safety of erenumab, which is another anti-CGRP antibody^17,32,37^. The present study is the first network analysis evaluating the efficacy and safety of fremanezumab and the first study to assess the efficacy of different fremanezumab medication strategies.

Although we were able to overcome some methodological limitations in pairwise meta-analysis for synthesis by applying the consistency model, the analysis had some limitations. First, the types of migraines could not be stratified in our synthesis; separation of chronic migraine from episodic migraine data would not permit the application of the consistency model. Therefore, future research should assess the efficacy of fremanezumab in treating either chronic or episodic migraines. Second, sex is a critical factor in the development of migraines; however, data could not be stratified according to sex. The differences in the effects of fremanezumab on migraines in men and women have not been investigated. We encourage future studies to investigate the patterns of response to treatment with fremanezumab in men and women.

Finally, the current considerations in the development of anti-CGRP antibodies are identity, expression pattern, and function of CGRP receptors, which affect the safety and efficacy of these antibodies. Although CGRP is capable of activating multiple receptors, it binds with high affinity to two receptors, the CGRP receptor and Amylin-1 (AMY1) receptor, which are considered more physiological relevance than other receptors and play a central role in the molecular pathophysiology of the trigeminovascular system^38,39^. The function of anti-CGRP antibodies may exhibit different profiles in blocking the cross-talk between the peptides and their receptors^40^. For instance, erenumab does not block the AMY1 receptor but only the CGRP receptor^38^. Therefore, further studies are warranted to investigate the therapeutic differences in these anti-CGRP antibodies targeting various CGRP-relevant receptors.

Our findings revealed that fremanezumab 675 mg exhibited the highest 50% reduction rate, whereas 225 mg monthly yielded the highest 75% reduction rate. The long-term effects of these treatment strategies warrant further investigation. Furthermore, we observed a trend toward higher incidences of serious adverse events at higher dosages. This trend may raise clinical concern (RR 2.09) despite being nonsignificant. Therefore, future studies should further clarify the dosing efficacy and evaluate the long-term safety of treatment with fremanezumab.

## Methods

The protocol for the present study was modified from our research on anti-CGRP treatments for migraines (PROSPERO: CRD42018118063). Therefore, the PRISMA guidance was followed for all processes, including evidence selection, quality assessment, evidence synthesis, and research reporting^41^. Three modifications were made to the previous protocol. First, terms relevant to fremanezumab were used in the search. Second, a contrast-based network meta-analysis was used for quantitative synthesis. Third, the occurrence of adverse events was also examined because the primary aim of the study was to improve the practical understanding of fremanezumab treatment strategies.

### Eligibility criteria and evidence selection

The eligibility criteria for evidence selection were defined before the search. The primary inclusion criteria for research studies were as follows: (a) recruited patients with migraine and (b) treated patients with fremanezumab. Therefore, PubMed, Embase, and Web of Science were searched using terms relevant to migraine and fremanezumab in free-text, medical subject headings, and abbreviations. The search strategy was not restricted by language or date of publication.

Two authors (IHH and PCW) completed the search before February 2020 (Supplementary File S17) and independently identified references by screening titles and abstracts. The full texts of the selected references were then retrieved. After reviewing the full text, the authors excluded articles based on the following exclusion criteria: (a) non-RCTs; (b) studies on healthy individuals, patients without migraines, or mixed patients with general headache and migraine without a stratified analysis; (c) studies in which patients were not treated with fremanezumab or treated with combination therapy; and (d) gray literature without details. In case of disagreement between the two authors regarding evidence selection, a third experienced author (YNK) participated in the process, and a consensus was reached after discussion.

### Data extraction and quality assessment

All included RCTs were reviewed by the two authors (IHH and PCW) who individually extracted study design, trial characteristics, and outcome data. The characteristics included the trial registry number, location, study period, medication strategy, mean age, sex, and type of migraine. Outcomes were the cumulative 50% reduction rate, cumulative 75% reduction rate, reduction in the number of migraine days, treatment-related adverse events, and serious adverse events. Most of these outcomes (i.e., number of events, cumulative 50% reduction rate, cumulative 75% reduction rate, treatment-related adverse events, and serious adverse events) were presented as rates. The reduction in the number of migraine days was presented as a continuous result variable; mean and standard deviation were extracted along with the total sample size. The quality of the included RCTs was assessed using the Cochrane Risk of Bias Tool.

### Data synthesis and analysis

A contrast-based network meta-analysis was employed for quantitative synthesis because the efficacy and safety of several fremanezumab medication strategies have been tested. Network meta-analysis is a statistical technique used to test numerous competing interventions for a condition by combining direct and indirect evidence^42^. In addition to dosage, various frequencies and strategies of treatment with fremanezumab have been tested for migraine prevention in RCTs. However, some medication strategies were not directly compared in the previous evidence. Therefore, a network meta-analysis is more appropriate than the traditional pairwise meta-analysis, meta-regression, and dose–response meta-analysis. There are some debate concerning the selection of model of network meta-analysis. In our study,a contrast-based network meta-analysis (frequentist model) was used because relevant studies have indicated that pooled estimates in frequentist models are similar to those in a Bayesian model^43,44^, and the frequentist approach is easily understood and commonly applied in the statistical field^45^. In contrast with the Bayesian model, the frequentist model does not require any prior for carrying out network meta-analysis. Therefore, the present study did not set prior parameters for the analysis. Moreover, the present study satisfied the core assumptions of network meta-analysis^46^. For transitivity, this synthesis defined a specific PICO framework and maintained similarity among trials by including RCTs after a comprehensive search^42,46^. The available evidence formed a closed loop for the consistency test^47^, and manifested transitivity statistically^48^.

Cases of cumulative 50% reduction, cumulative 75% reduction, treatment-related adverse event, serious adverse event, and the total cases in each group were the main parameters for performing the network meta-analysis. This study pooled data by fitting the network meta-analysis model using the “*network*” package in STATA version 14 for Microsoft Windows (Texas, USA). To fit the consistency model, the “*network*” package estimated the direct relative effects using a head-to-head meta-analysis method before synthesizing the direct relative effects to estimate the network relative effects^49^. The relative effects were mainly estimated based on the log RR and standard error of the log RR in the network meta-analysis. Therefore, RR was calculated for the cumulative 50% reduction rate, cumulative 75% reduction rate, treatment-related adverse events, and serious adverse events by using the “eform” option^49^. The MD was estimated for the reduction in the number of migraine days. Our results were presented as effect size and 95% CI. If a 95% CI crosses a null value, the pooled estimate was not significantly different between treatments. The SUCRA technique was used to help identify significant results in a consistency model for clinical practice^50^. Therefore, the SUCRA was used to determine the effects of medication strategies using fremanezumab. By ranking the probability of each comparator among the most effective treatments, the SUCRA was used to create a hierarchy indicating the optimal medication strategy. A SUCRA value of 0 for a fremanezumab medication strategy indicates the least favorable strategy, whereas a strategy may be the most effective treatment for migraine and adverse event prevention if its SUCRA value is 100. However, the optimal strategy of fremanezumab cannot be identified based on the SUCRA of each outcome alone. Balancing between effectiveness and adverse events is crucial for clinicians when choosing a fremanezumab treatment for migraines. Efficacy and safety are typical outcomes for recommending treatment in clinical practice. Two-dimensional plots and clustering methods are appropriate techniques to classify potential sets of treatments because cluster analysis group observations are based on certain features and degrees of association between members of the same group and different groups^51^. We further constructed SUCRA cluster plots of the cumulative 50% reduction rate, cumulative 75% reduction rate, and treatment-related adverse events.

Each network meta-analysis was assessed for inconsistencies and publication bias in the pooled estimates. Inconsistencies were identified using the loop inconsistency test, and publication biases were detected by producing a funnel plot with Egger’s test. A *P* value of < 0.05 on the Egger’s test denoted a publication bias, indicating that the pooled estimate should be interpreted with caution. If Egger’s test failed to detect a small study effect because of limited formation and variance in the comparison-adjusted funnel plot, an alternative method of Begg and Mazumdar was employed. All analyses were performed using STATA.

### Ethics approval and consent to participate

Not applicable.

## Supplementary information

Supplementary Information.

## Data Availability

All data generated or analysed in this study can be found in the randomized controlled trials we included in this synthesis.

## Abbreviations

50%CRR: Cumulative 50% reduction rate
75%CRR: Cumulative 75% reduction rate
Anti-CGRP: Anti-calcitonin gene-related peptide
CI: Confidence intervals
MD: Mean difference
RCT: Randomized clinical trial
RR: Risk ratio
S675mg: Single high dose of 675 mg
SUCRA: Surface under the cumulative ranking
