# Supplementary material for: Optimal treatment strategy of fremanezumab in migraine prevention: a systematic review with network meta-analysis of randomized clinical trials
Source: Sci Rep. 2020 Oct 29;10:18609. doi: 10.1038/s41598-020-75602-8 (PMC7596067; doi:10.1038/s41598-020-75602-8)
Supplement: Supplementary file 1 — Supplementary Information. [file 41598_2020_75602_MOESM1_ESM.pdf]

Optimal treatment strategy of fremanezumab in migraine prevention:
A systematic review with network meta-analysis of randomized clinical trials

I-Hsin Huang, M.D., Po-Chien Wu, M.D., Ya-Han Lee, MS, Pharm., Yi-No Kang, Consultant

Supplementary Files

Supplementary File S1. Risk of bias

Supplementary File S2. Surface under the cumulative ranking curve for cumulative 50% reduction rate

Supplementary File S3. Loop inconsistency test for cumulative 50% reduction rate

Supplementary File S4. Small study effect test for cumulative 50% reduction rate

Supplementary File S5. Surface under the cumulative ranking curve for cumulative 75% reduction rate

Supplementary File S6. Loop inconsistency test for cumulative 75% reduction rate

Supplementary File S7. Small study effect test for cumulative 75% reduction rate

Supplementary File S8. Surface under the cumulative ranking curve for reduction of migraine days

Supplementary File S9. Loop inconsistency test for reduction of migraine days

Supplementary File S10. Small study effect test for reduction of migraine days

Supplementary File S11. Surface under the cumulative ranking curve for treatment-related adverse events

Supplementary File S12. Loop inconsistency test for treatment-related adverse events

Supplementary File S13. Small study effect test for treatment-related adverse events

Supplementary File S14. Surface under the cumulative ranking curve for serious adverse events

Supplementary File S15. Loop inconsistency test for serious adverse events

Supplementary File S16. Small study effect test for serious adverse events

Supplementary File S17. Search strategy

Supplementary File S1
Risk of bias

Supplementary File S1

Risk of bias summary

Study	NCT 02629861	NCT 02621931	NCT 02021773	NCT 02025556	NCT 03308968
Sequence generation	Low risk	Low risk	Low risk	Low risk	Low risk
Allocation concealment	Low risk	Low risk	Low risk	Low risk	Low risk
Blinding of participants and personnel	Low risk	Low risk	Low risk	Low risk	Low risk
Blinding of outcome assessment	Low risk	Low risk	Low risk	Low risk	Low risk
Incomplete outcome data	Low risk	Low risk	Low risk	Low risk	Low risk
Selective reporting	Low risk	Low risk	Low risk	Low risk	Low risk
Other bias	High risk	High risk	High risk	High risk	High risk

Supplementary Files S2 to S4

Results of cumulative 50% reduction rate

Supplementary File S2

Surface under the cumulative ranking curve for cumulative 50% reduction rate

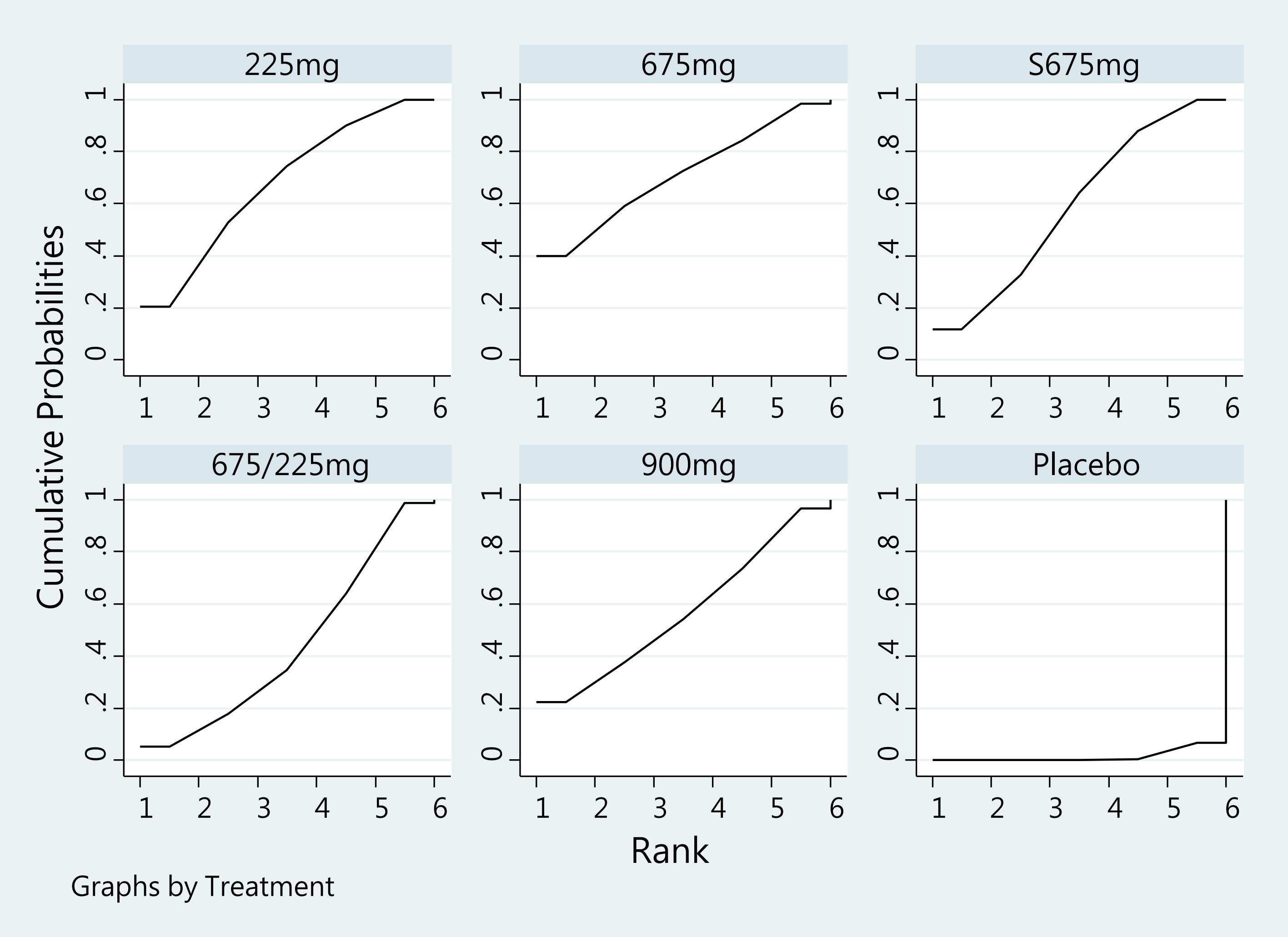

Treatment	SUCRA	PrBest	MeanRank
Placebo	1.4	0.0	5.9
225mg	67.6	20.5	2.6
S675mg	59.3	11.8	3.0
675/225mg	44.0	5.3	3.8
675mg	70.9	40.0	2.5
900mg	56.8	22.4	3.2

Supplementary File S3

Loop inconsistency test for cumulative 50% reduction rate

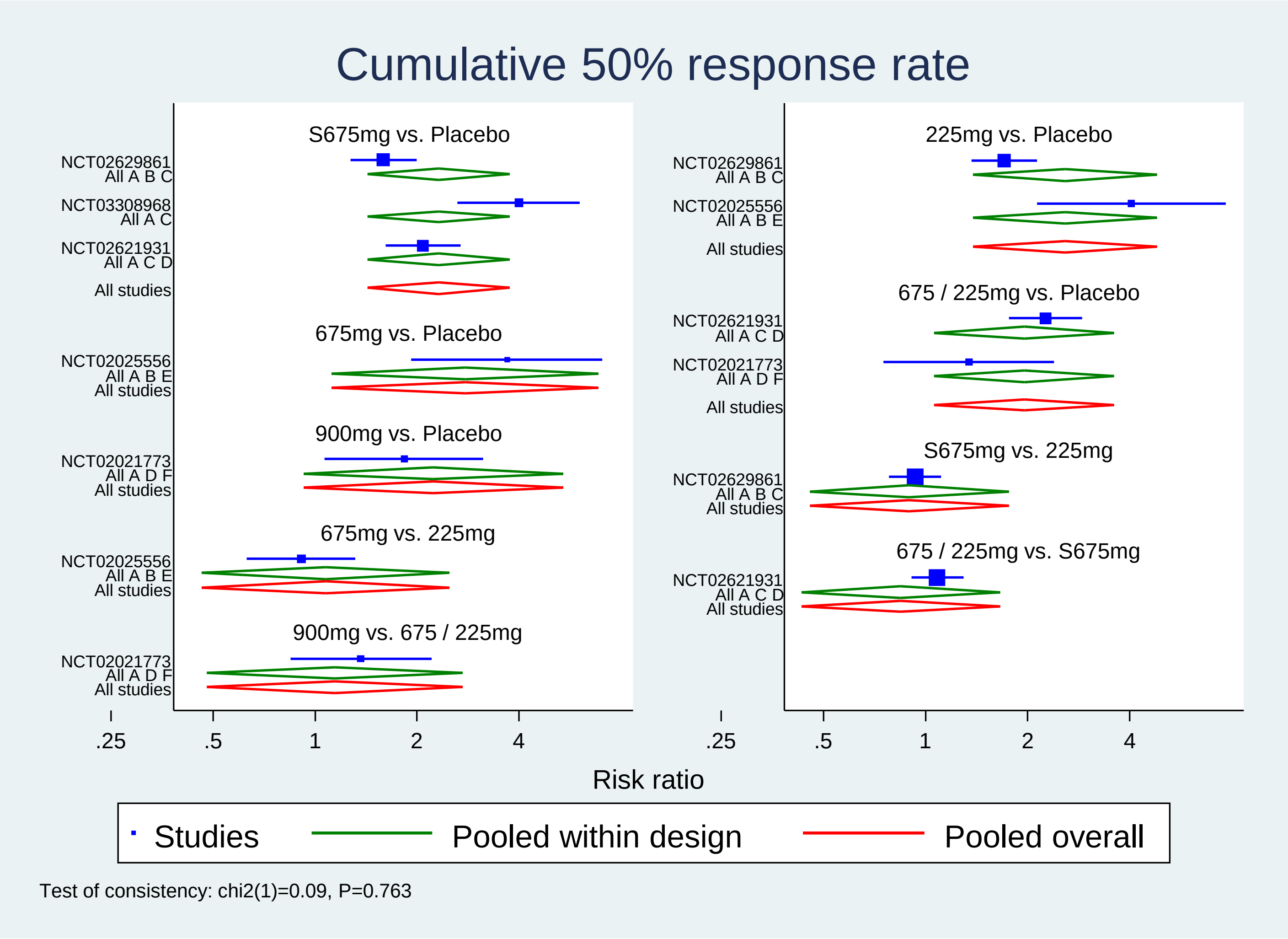

Supplementary File S4

Small study effect test for cumulative 50% reduction rate

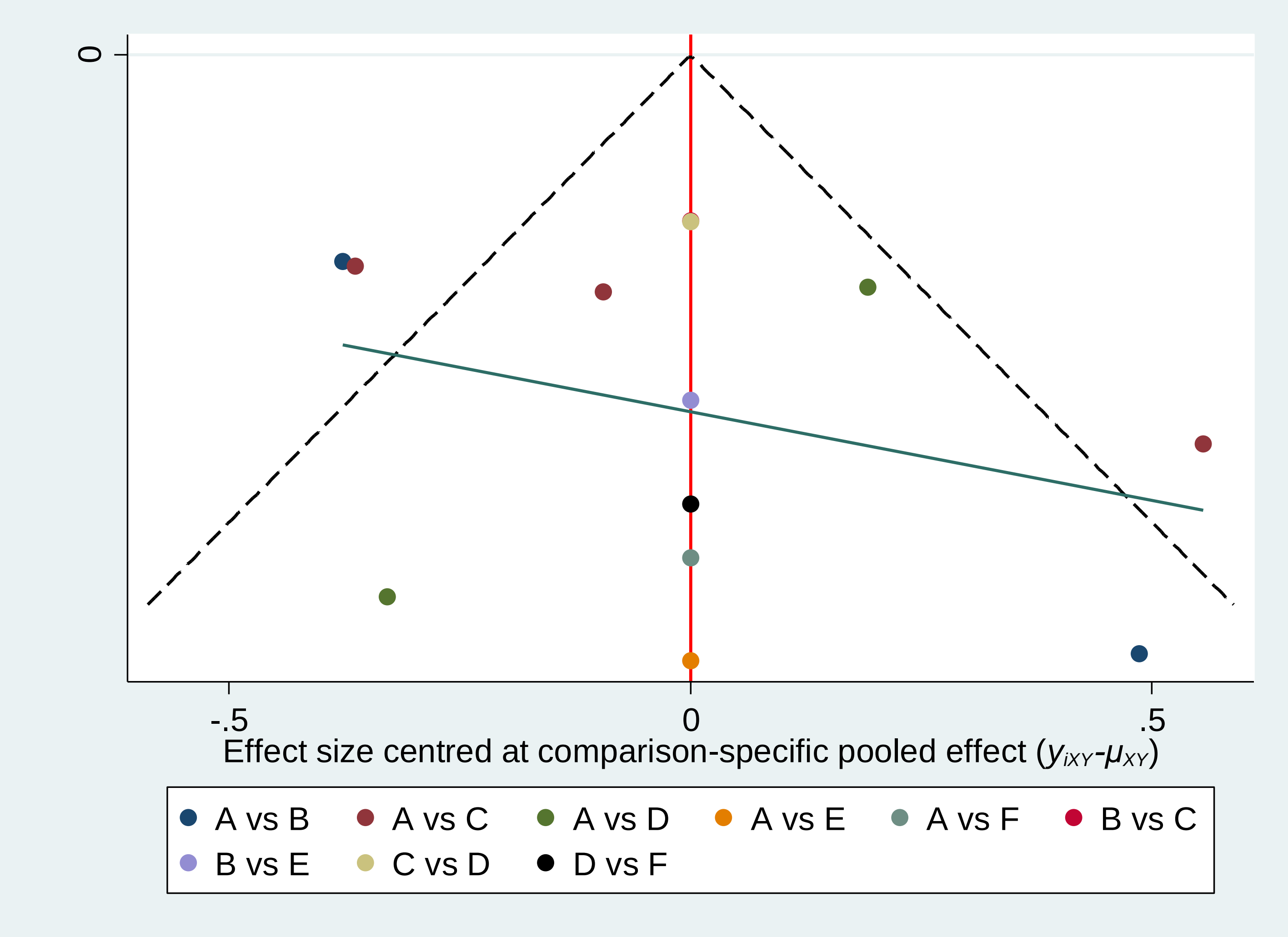

A, placebo; B, 225mg; C, S675mg; D, 675 / 225mg; E, 675mg; F, 900mg

```
Egger's test for small-study effects:
Regress standard normal deviate of intervention
effect estimate against its standard error

Number of studies =  13                          Root MSE      =   1.646
------------------------------------------------------------------------------
     Std_Eff |      Coef.   Std. Err.       t    P>|t|     [95% Conf. Interval]
-------------+----------------------------------------------------------------
       slope |  -.1911849   .1592369    -1.20   0.255    -.5416631    .1592932
        bias |   1.002743   1.108367     0.90   0.385    -1.436757    3.442243
------------------------------------------------------------------------------

Test of H0: no small-study effects          P = 0.385
```

Supplementary Files S5 to S7

Results of cumulative 75% reduction rate

Supplementary File S5

Surface under the cumulative ranking curve for cumulative 75% reduction rate

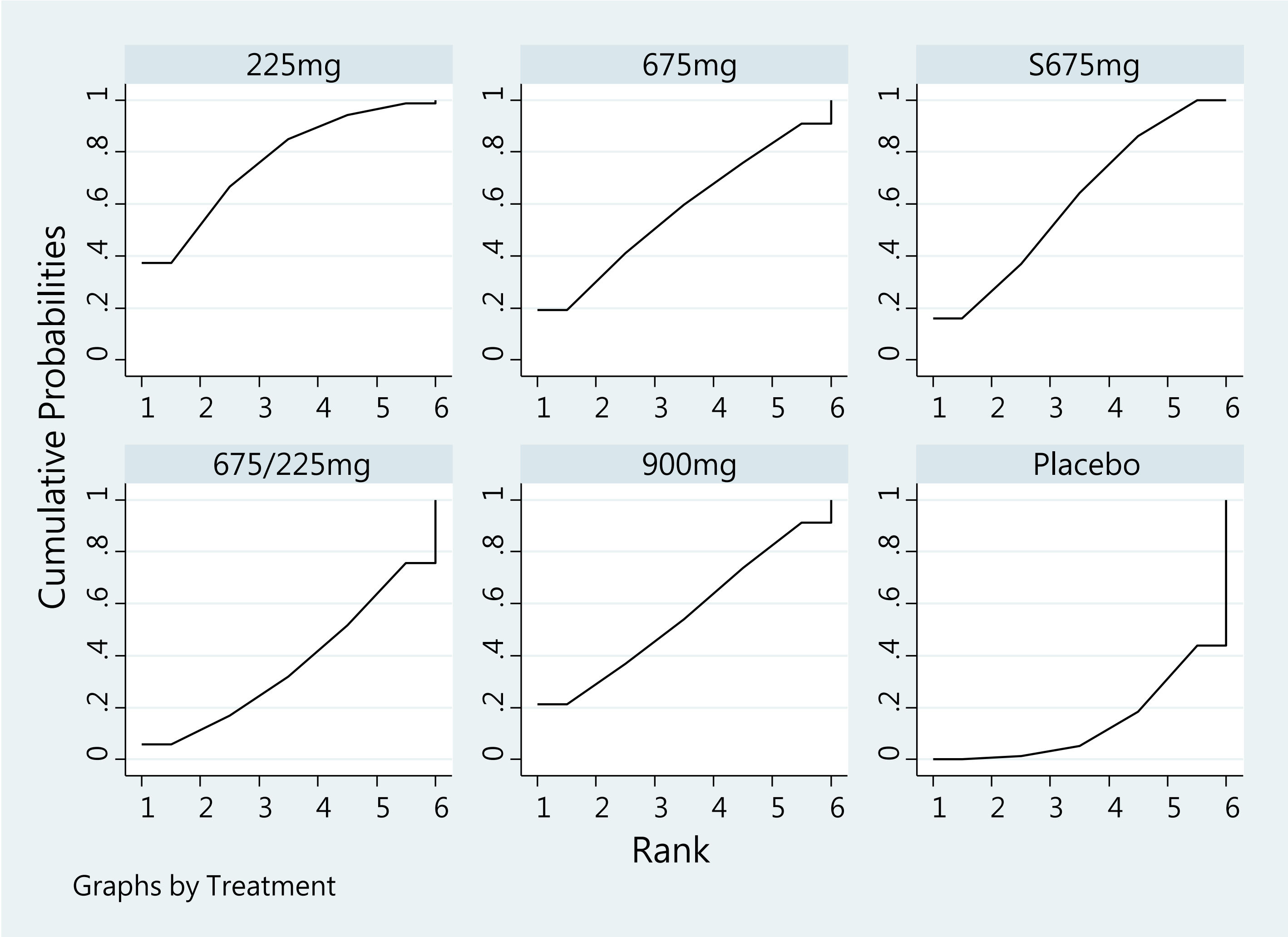

Treatment	SUCRA	PrBest	MeanRank
Placebo	13.7	0.0	5.3
225mg	76.4	37.4	2.2
S675mg	60.7	15.9	3.0
675/225mg	36.4	5.8	4.2
675mg	57.4	19.4	3.1
900mg	55.4	21.5	3.2

Supplementary File S6

Loop inconsistency test for cumulative 75% reduction rate

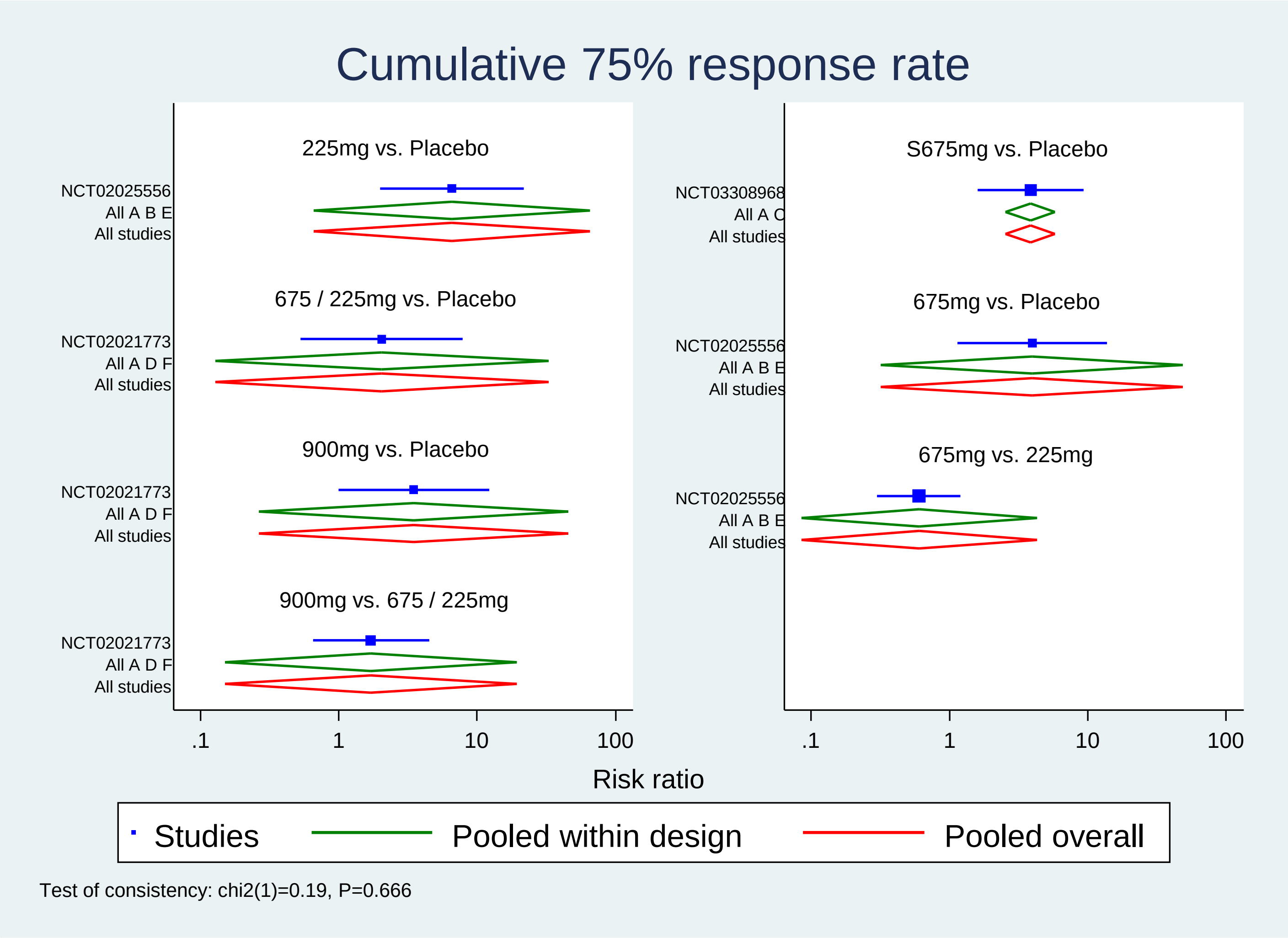

Supplementary File S7

Small study effect test for cumulative 75% reduction rate

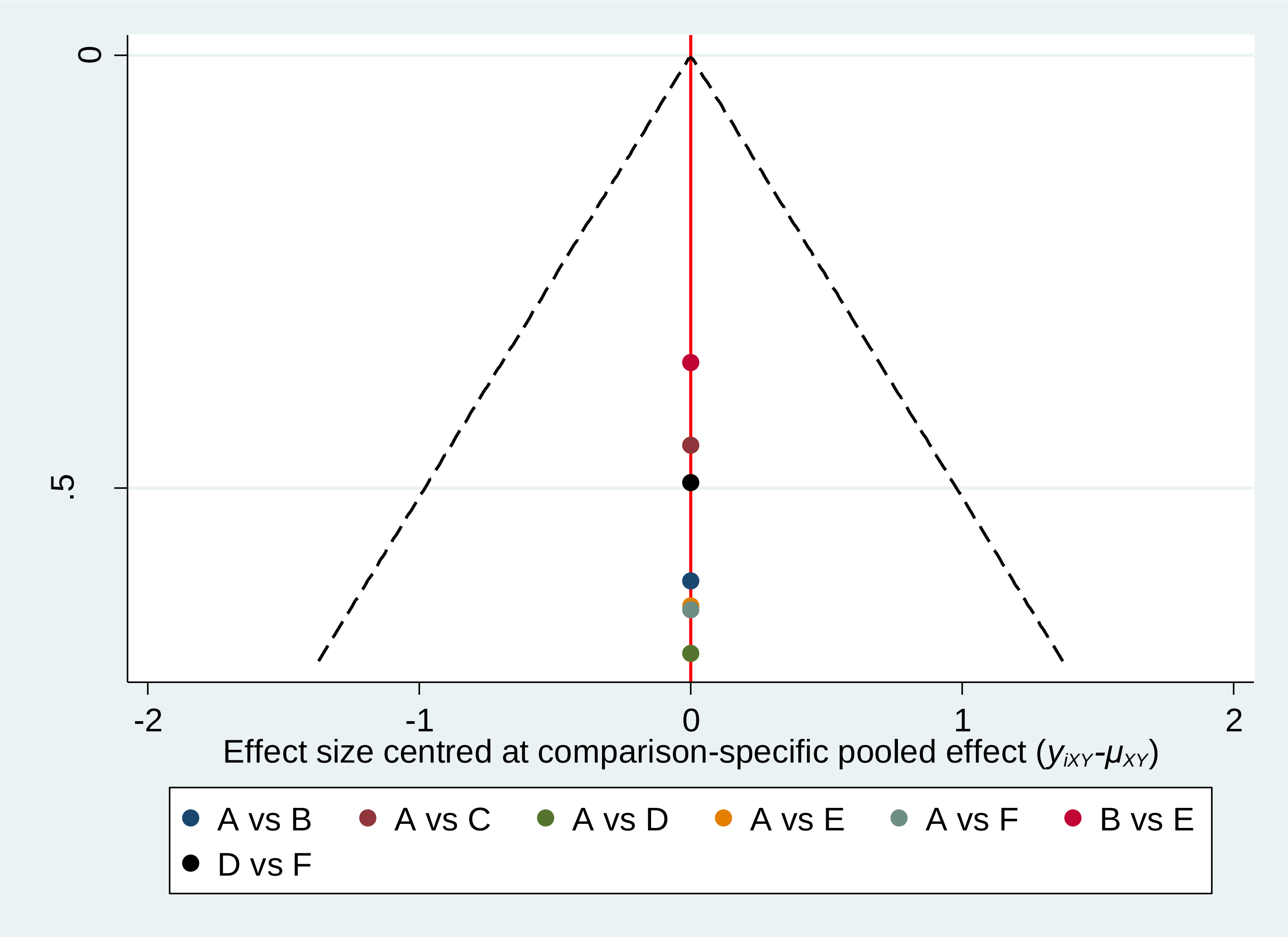

A, placebo; B, 225mg; C, S675mg; D, 675 / 225mg; E, 675mg; F, 900mg

```
Egger's test for small-study effects:
Regress standard normal deviate of intervention
effect estimate against its standard error

Number of studies =  7                                  Root MSE      =       0
------------------------------------------------------------------------------
     Std_Eff |      Coef.   Std. Err.      t    P>|t|     [95% Conf. Interval]
-------------+----------------------------------------------------------------
       slope |  (dropped)
        bias |  (dropped)
------------------------------------------------------------------------------

Begg's test for small-study effects:
Rank correlation between standardized intervention effect and its standard error

  adj. Kendall's Score (P-Q) =       0
          Std. Dev. of Score =    9.42 (corrected for ties)
           Number of Studies =       7
                          z  =   -0.11 (continuity corrected)
                   Pr > |z|  =   1.000 (continuity corrected)
```

Supplementary Files S8 to S10

Results of reduction of migraine days

Supplementary File S8

Surface under the cumulative ranking curve for reduction of migraine days

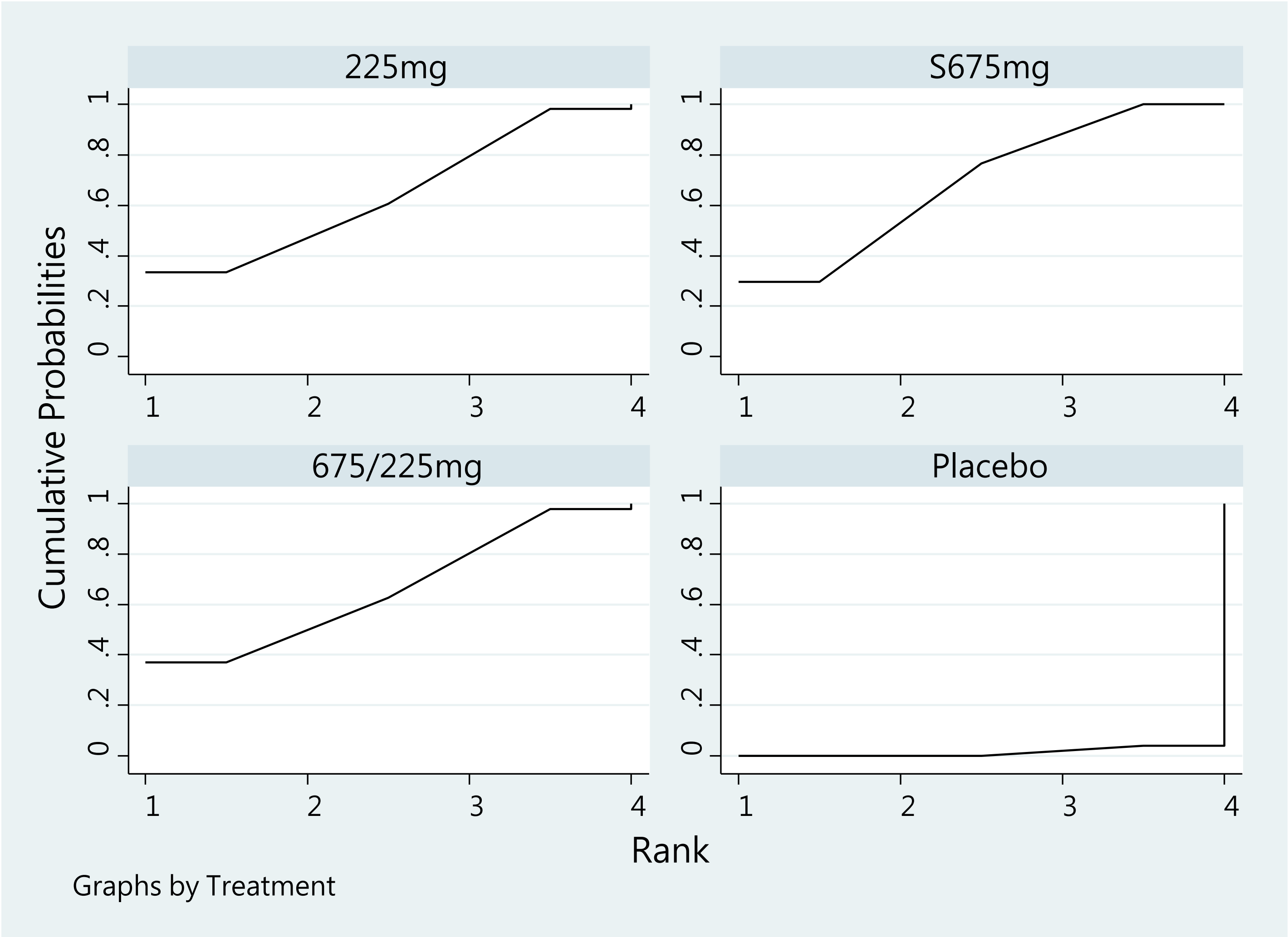

Treatment	SUCRA	PrBest	MeanRank
Placebo	1.4	0.0	4.0
225mg	64.1	33.4	2.1
S675mg	68.7	29.7	1.9
675/225mg	65.8	36.9	2.0

Supplementary File S9

Loop inconsistency test for reduction of migraine days

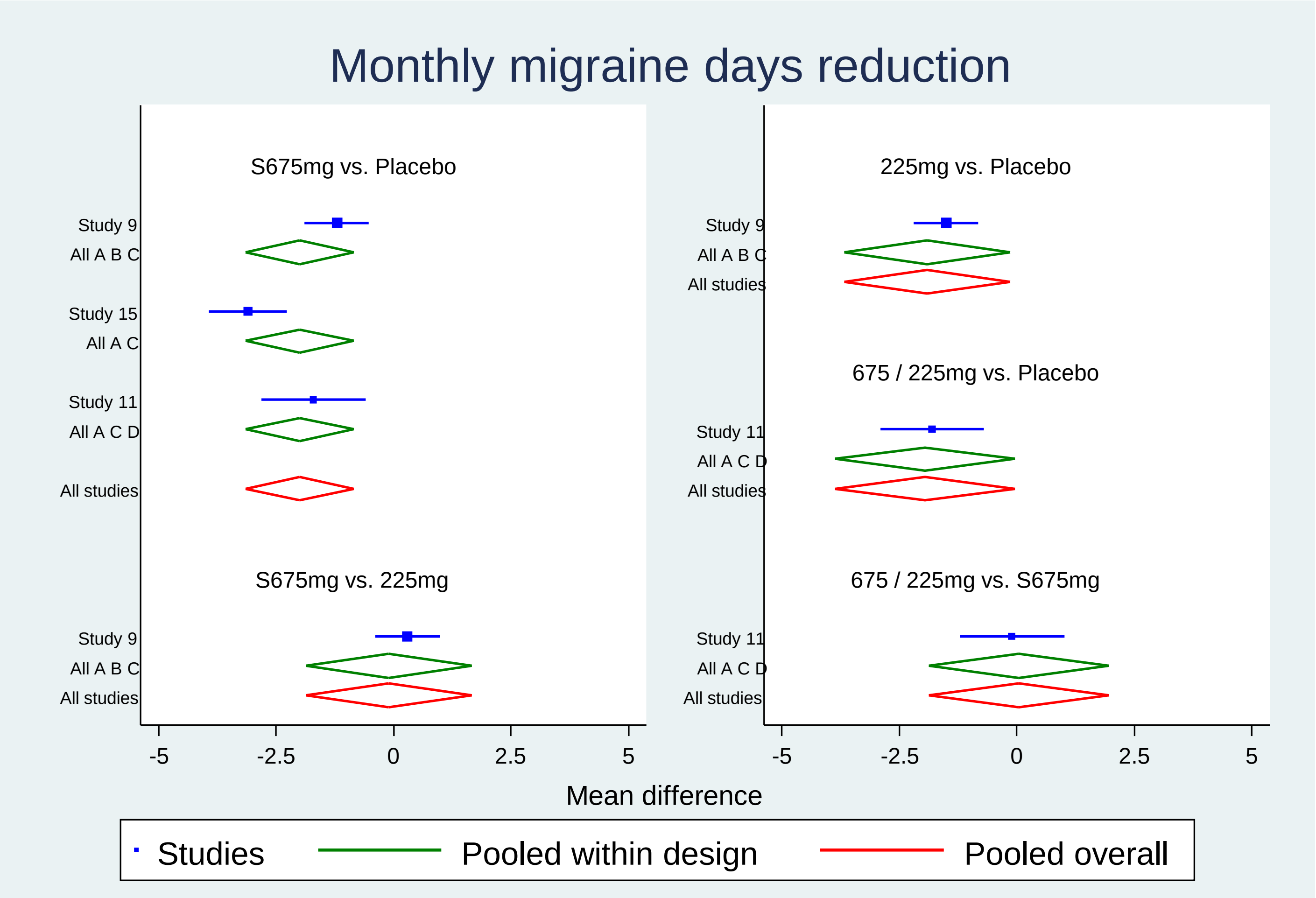

Test of consistency: chi2(1)=0.00, P=0.961

Supplementary File S10

Small study effect test for reduction of migraine days

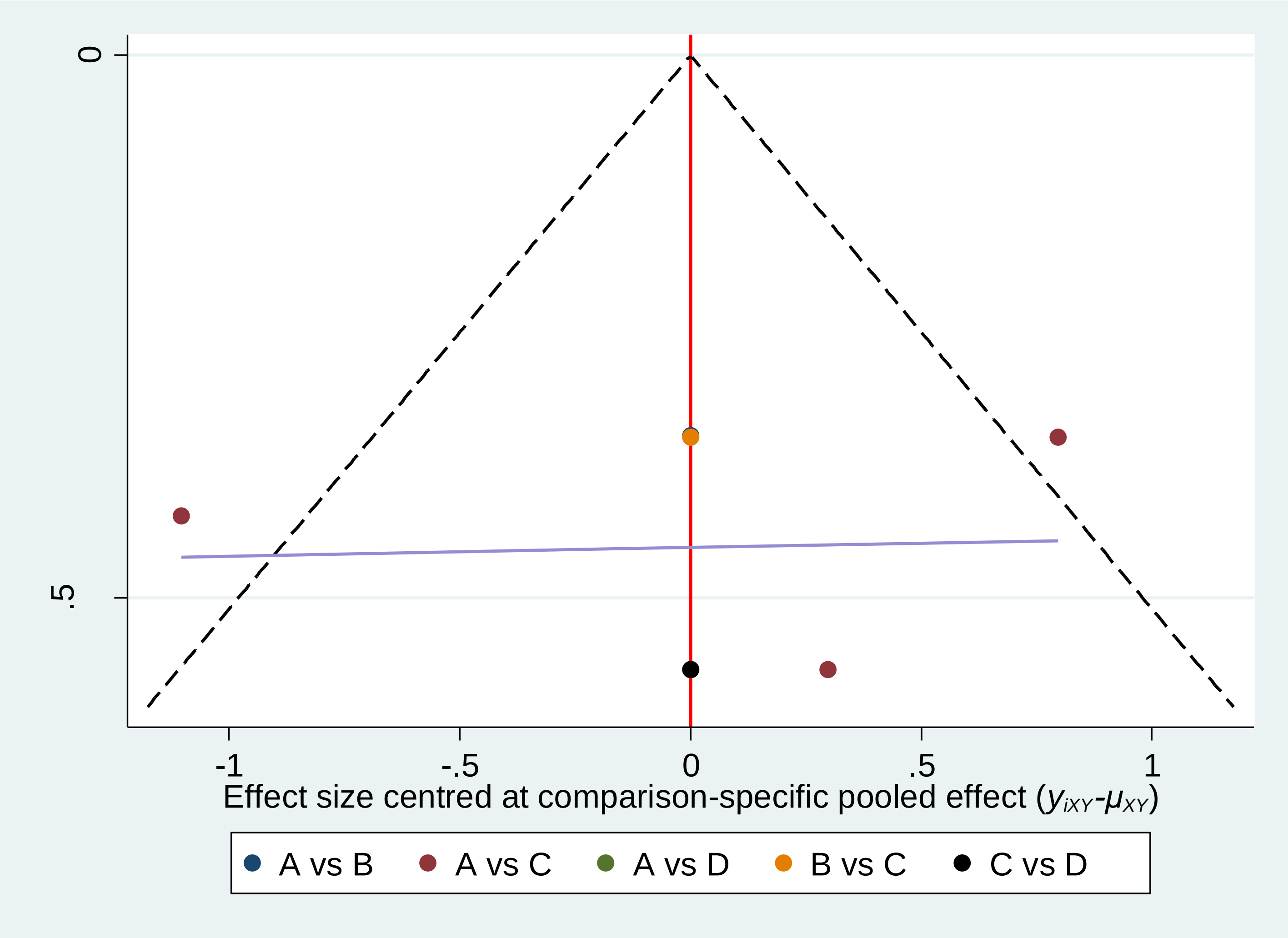

A, placebo; B, 225mg; C, S675mg; D, 675 / 225mg

```
Egger's test for small-study effects:
Regress standard normal deviate of intervention
effect estimate against its standard error

Number of studies =  7                          Root MSE      =   1.536
------------------------------------------------------------------------------
     Std_Eff |      Coef.   Std. Err.      t    P>|t|     [95% Conf. Interval]
-------------+----------------------------------------------------------------
       slope |   .4564213   1.160567     0.39   0.710    -2.526911    3.439754
        bias |  -1.029186    2.74852    -0.37   0.723     -8.09448    6.036109
------------------------------------------------------------------------------

Test of H0: no small-study effects          P = 0.723
```

Supplementary Files S11 to S13

Results of treatment-related adverse events

Supplementary File S11

Surface under the cumulative ranking curve for treatment-related adverse events

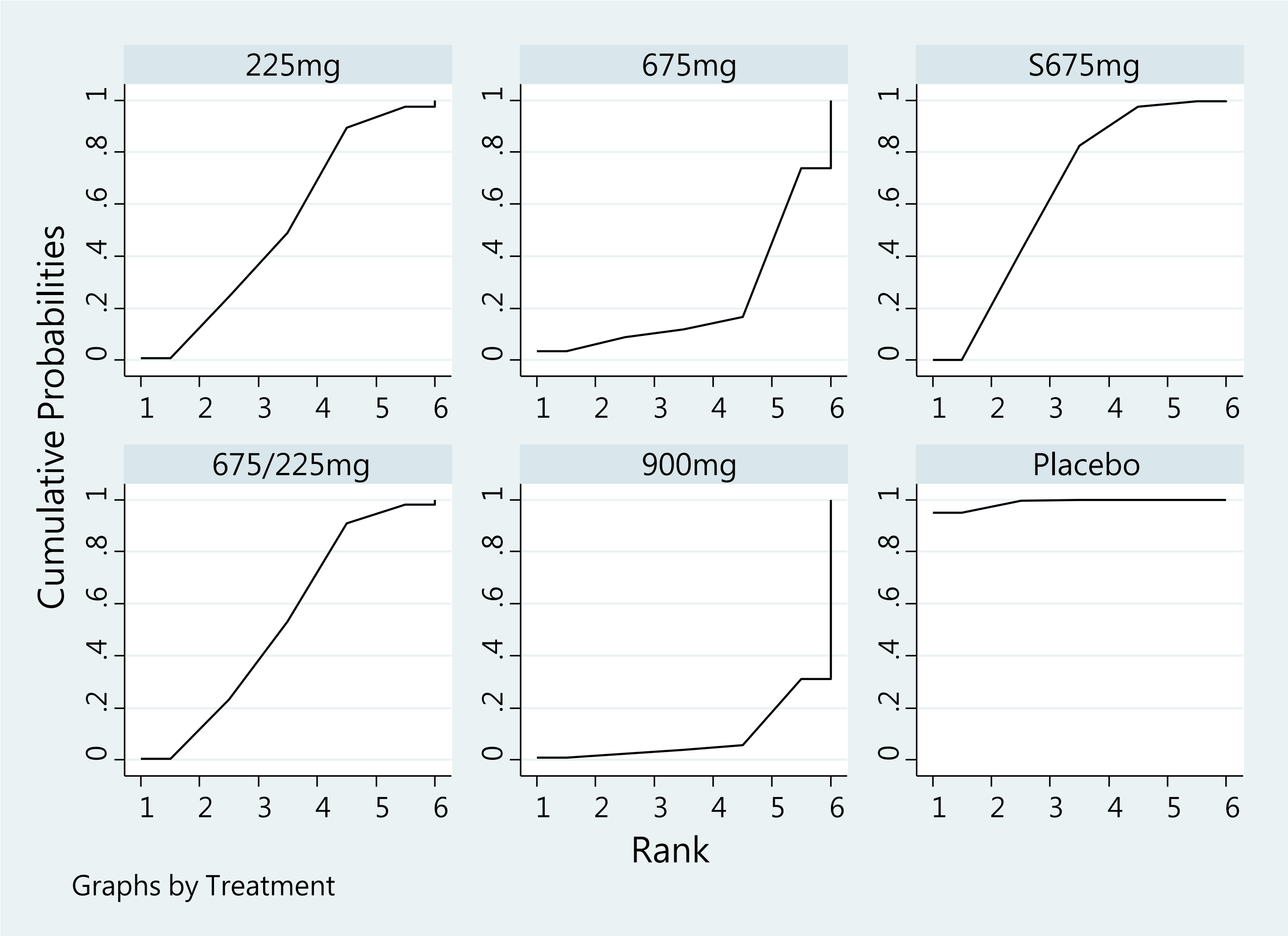

Treatment	SUCRA	PrBest	MeanRank
Placebo	98.9	95.0	1.1
225mg	52.2	0.7	3.4
S675mg	64.4	0.2	2.8
675/225mg	53.1	0.3	3.3
675mg	22.9	3.3	4.9
900mg	8.5	0.6	5.6

Supplementary File S12

Loop inconsistency test for treatment-related adverse events

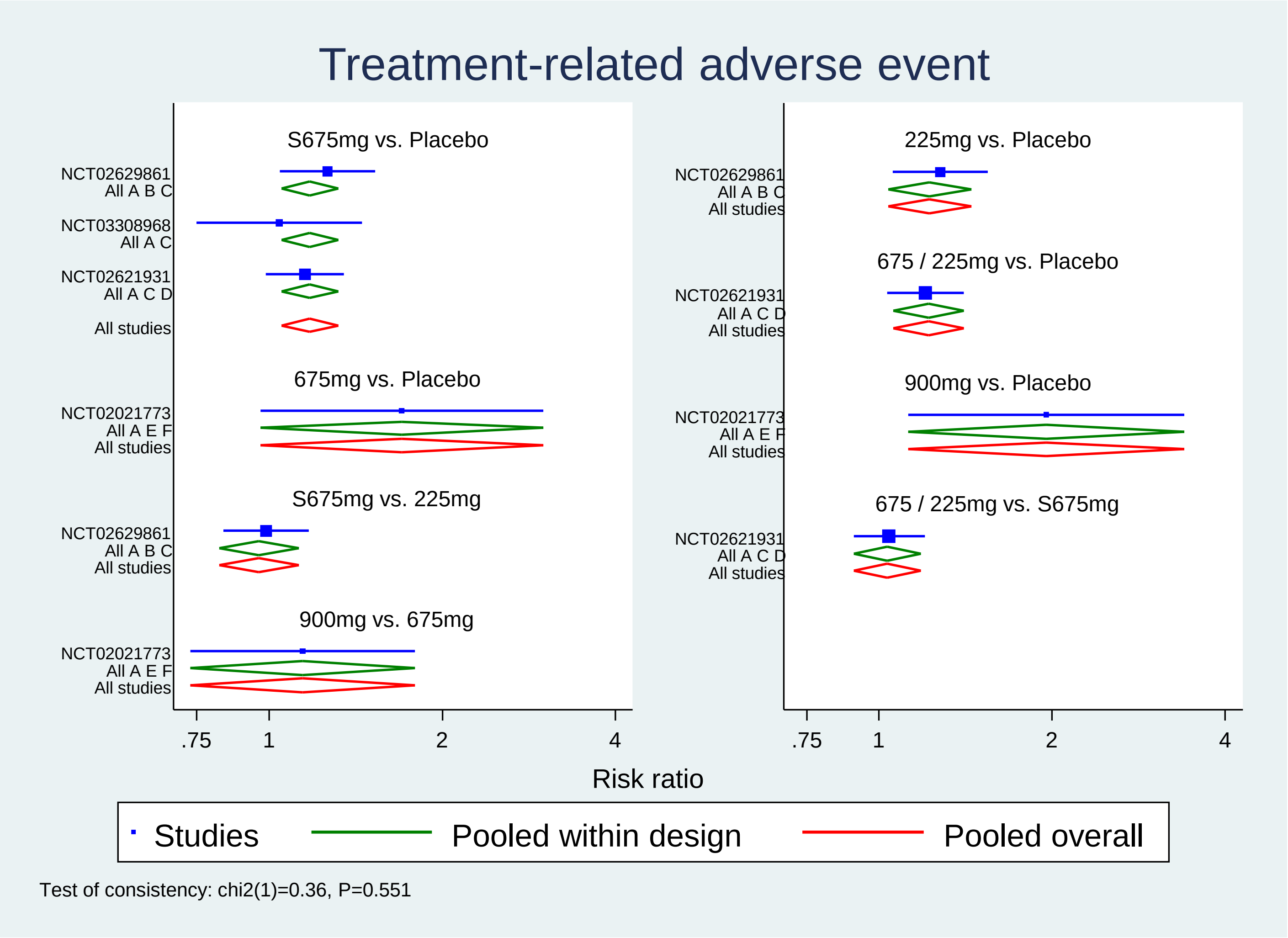

Supplementary File S13

Small study effect test for treatment-related adverse events

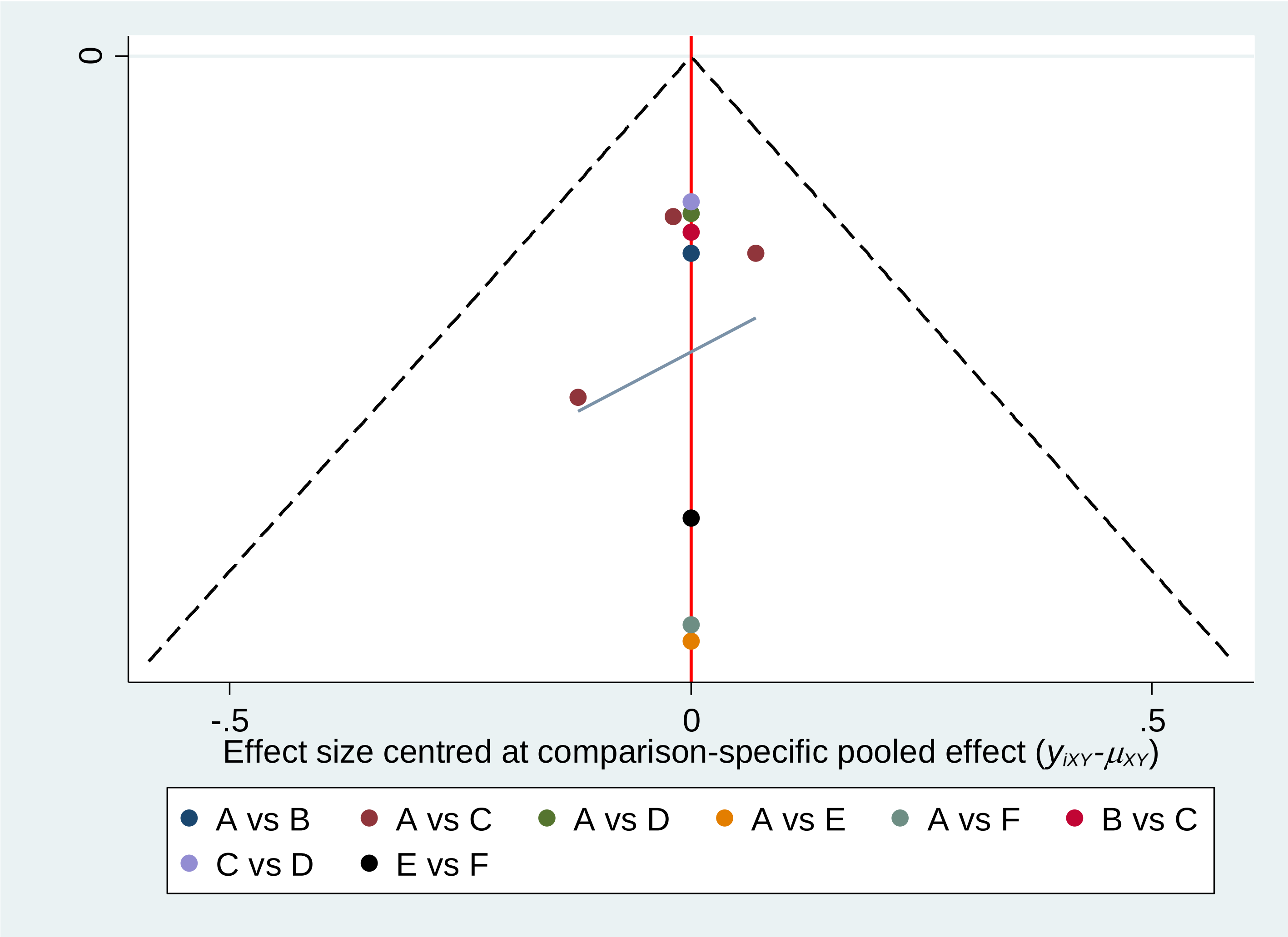

A, placebo; B, 225mg; C, S675mg; D, 675 / 225mg; E, 675mg; F, 900mg

Egger's test for small-study effects:
Regress standard normal deviate of intervention
effect estimate against its standard error

Number of studies = 10 Root MSE = .3643

Std_Eff	Coef.	Std. Err.	t	P>|t|	[95% Conf.	Interval]
slope	.0145499	.0295754	0.49	0.636	-.0536512	.082751
bias	-.1537801	.2861271	-0.54	0.606	-.8135905	.5060302

Test of H0: no small-study effects P = 0.606

Supplementary Files S14 to S16

Results of serious adverse events

Supplementary File S14

Surface under the cumulative ranking curve for serious adverse events

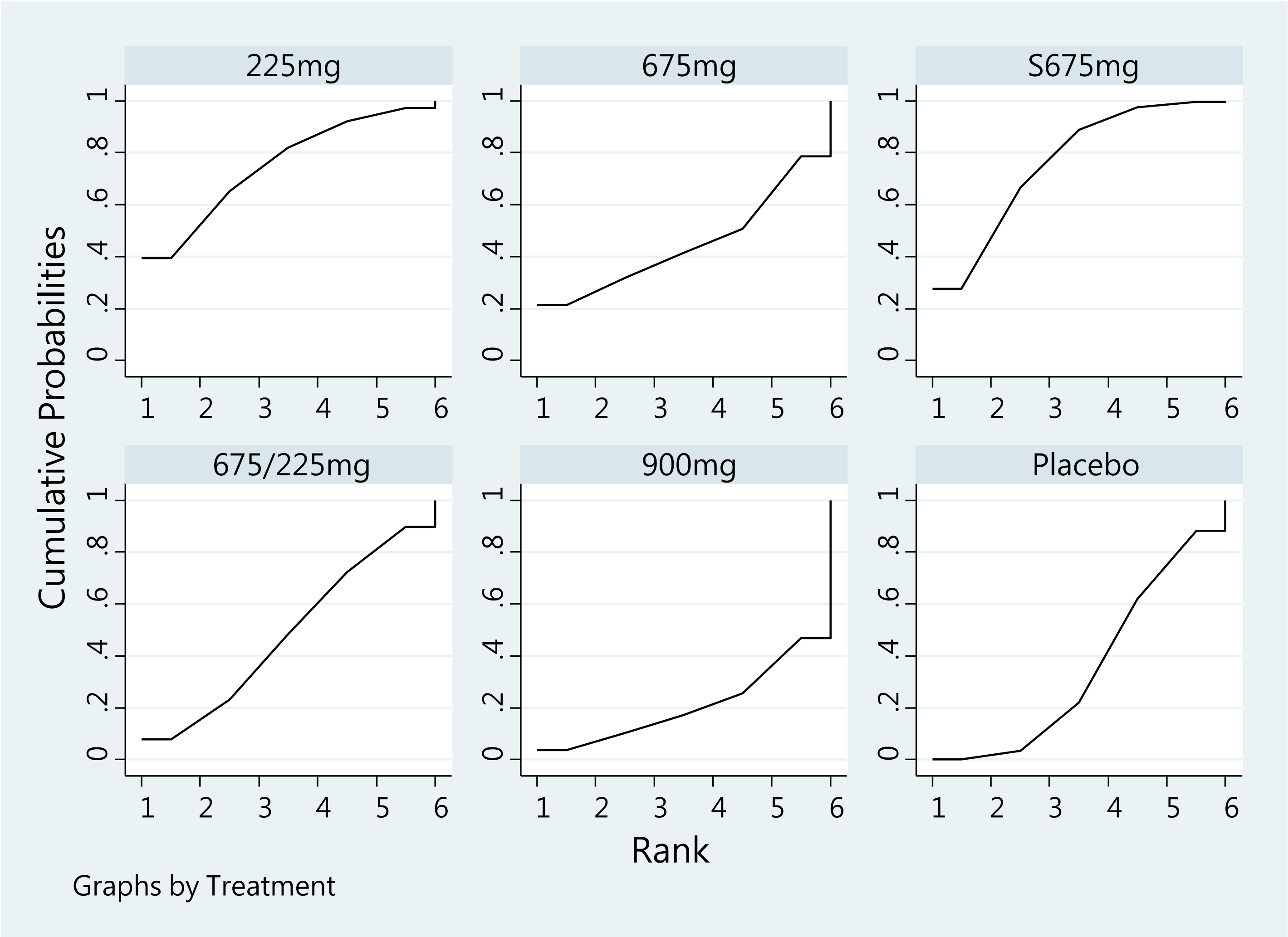

Treatment	SUCRA	PrBest	MeanRank
Placebo	35.1	0.2	4.2
225mg	75.1	39.3	2.2
S675mg	76.0	27.6	2.2
675/225mg	48.3	7.9	3.6
675mg	44.8	21.3	3.8
900mg	20.7	3.7	5.0

Supplementary File S15

Loop inconsistency test for serious adverse events

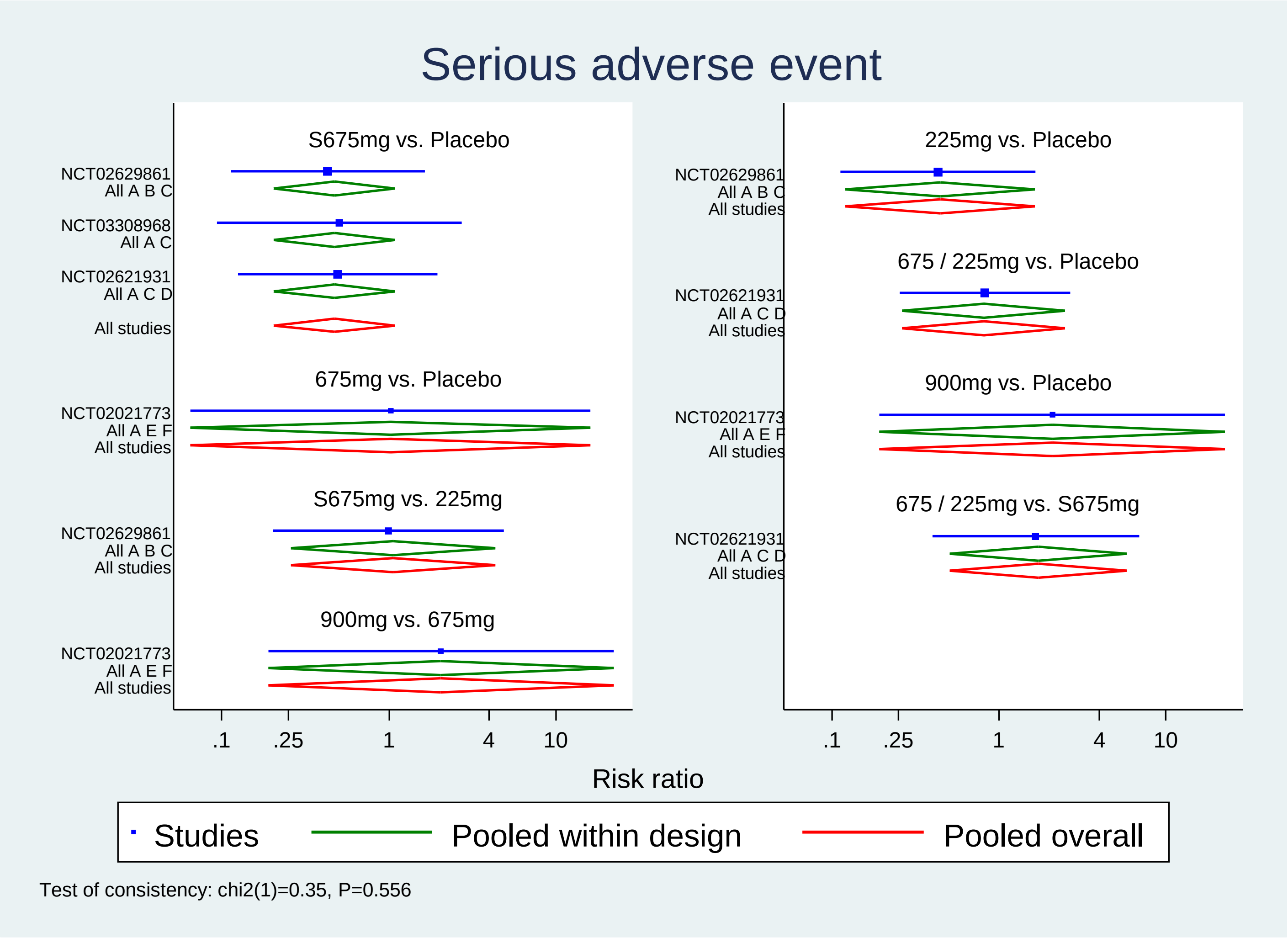

Supplementary File S16

Small study effect test for serious adverse events

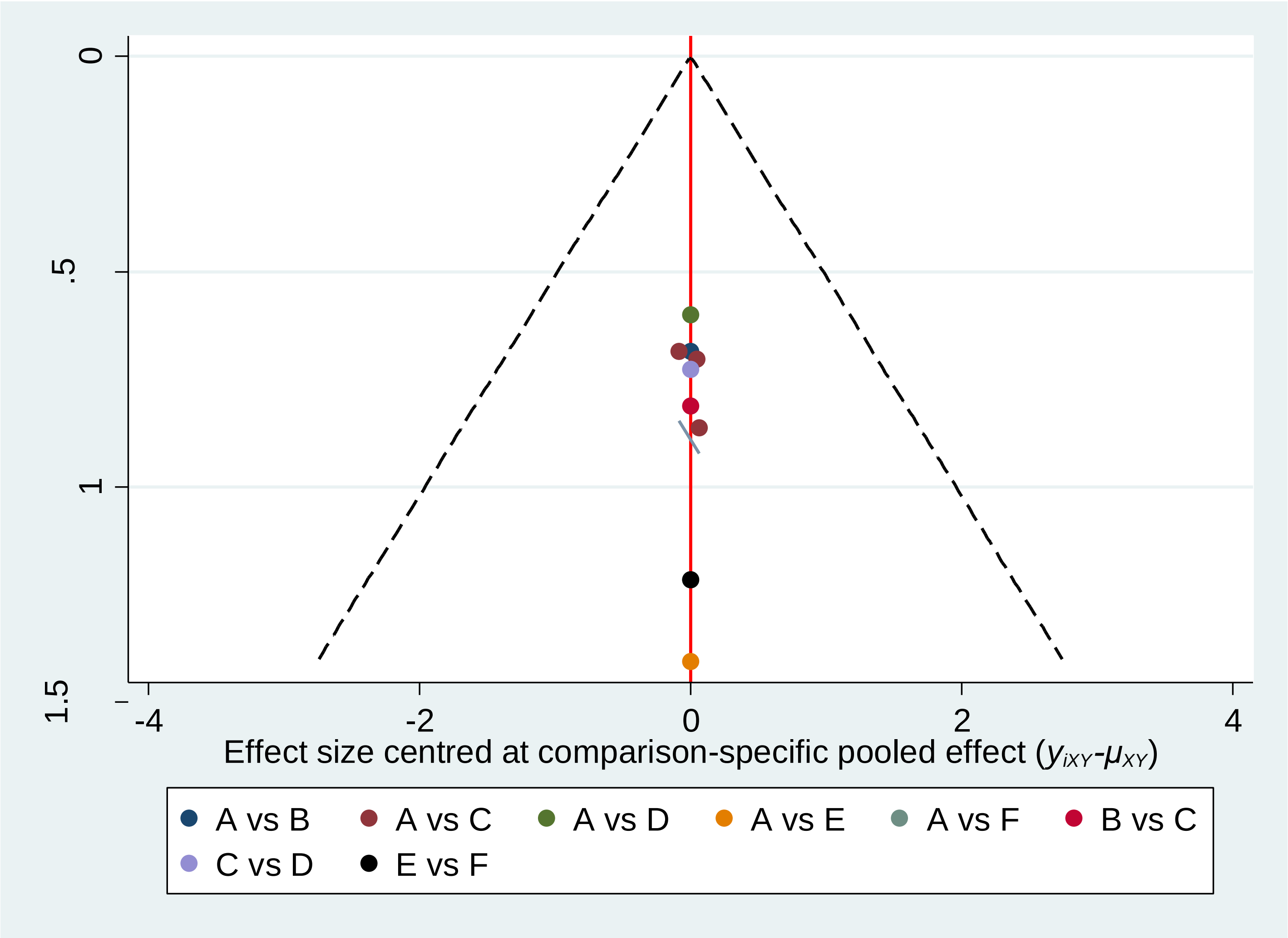

A, placebo; B, 225mg; C, S675mg; D, 675 / 225mg; E, 675mg; F, 900mg

```
Egger's test for small-study effects:
Regress standard normal deviate of intervention
effect estimate against its standard error

Number of studies =  10                        Root MSE      =   .0578
------------------------------------------------------------------------------
     Std_Eff |      Coef.   Std. Err.      t    P>|t|     [95% Conf. Interval]
-------------+----------------------------------------------------------------
       slope |   -.022378   .0591255    -0.38   0.715    -.1587215    .1139656
        bias |   .0289047   .0740071     0.39   0.706     -.141756    .1995654
------------------------------------------------------------------------------

Test of H0: no small-study effects          P = 0.706
```

Supplementary File S17

Search strategy

Supplementary File S17
Search strategy

Primary search steps:
#1. migraine
#2. fremanezumab
#3. tev 48125
#4. tev48125
#5. monoclonal anti-CGRP antibody
#6. monoclonal antibody to calcitonin gene-related peptide
#7. #2 OR #3 OR #4 OR #5 OR #6
#8. #1 AND #17

Final syntax in Embase:
('migraine'/exp OR migraine OR 'familial migraine' OR 'headache, migrainous' OR 'hemicrania' OR 'migraine' OR 'migraine disorders' OR 'status hemicranicus') AND ('monoclonal anti-cgrp antibody' OR 'monoclonal antibody to calcitonin gene-related peptide' OR 'fremanezumab'/exp OR fremanezumab OR 'fremanezumab' OR 'tev 48125' OR 'tev48125')

Final syntax in PubMed:
migraine AND (fremanezumab OR tev 48125 OR tev48125 OR monoclonal anti-CGRP antibody OR monoclonal antibody to calcitonin gene-related peptide)

Final syntax in Web of Science:
Topic: migraine AND Topic: (fremanezumab OR tev 48125 OR tev48125 OR monoclonal anti-CGRP antibody OR monoclonal antibody to calcitonin gene-related peptide)
